# Integrative Analysis of Cancer Omics Data for Prognosis Modeling

**DOI:** 10.3390/genes10080604

**Published:** 2019-08-09

**Authors:** Shuaichao Wang, Mengyun Wu, Shuangge Ma

**Affiliations:** 1 School of Life Sciences and Biotechnology, Shanghai Jiao Tong University, Shanghai 200240, China; 2 School of Statistics and Management, Shanghai University of Finance and Economics, Shanghai 200433, China; 3 Department of Biostatistics, Yale University, New Haven, CT 06520, USA

**Keywords:** multiple cancer types, integrative analysis, omics data, prognosis modeling

## Abstract

Prognosis modeling plays an important role in cancer studies. With the development of omics profiling, extensive research has been conducted to search for prognostic markers for various cancer types. However, many of the existing studies share a common limitation by only focusing on a single cancer type and suffering from a lack of sufficient information. With potential molecular similarity across cancer types, one cancer type may contain information useful for the analysis of other types. The integration of multiple cancer types may facilitate information borrowing so as to more comprehensively and more accurately describe prognosis. In this study, we conduct marginal and joint integrative analysis of multiple cancer types, effectively introducing integration in the discovery process. For accommodating high dimensionality and identifying relevant markers, we adopt the advanced penalization technique which has a solid statistical ground. Gene expression data on nine cancer types from The Cancer Genome Atlas (TCGA) are analyzed, leading to biologically sensible findings that are different from the alternatives. Overall, this study provides a novel venue for cancer prognosis modeling by integrating multiple cancer types.

## 1. Introduction

Cancer is one of the leading causes of death worldwide and has been posing extensive public concerns. In cancer studies, prognosis modeling is a critical step that greatly contributes to understanding cancer etiology, developing effective therapeutic methods, and improving life quality. Significant effort has been devoted to searching for prognostic factors, among which omics markers have important implications. For example, *EGFR* has been suggested as a strong prognostic indicator in multiple cancers, such as ovarian, cervical, and bladder cancers. Nicholson, et al. [1] reviewed over 200 studies and reported that relapse-free-interval or survival data are directly in relation to the increased EGFR levels in breast, gastric, colorectal, and many other cancers. Petitjean, et al. [2] found that the mutation of *TP53* has an impact on the prognosis of breast and several other cancers. Gao, et al. [3] used a Cox model to find that a high level of MMP-14 mRNA expression leads to a significantly shorter overall survival for breast cancer. Chiu, et al. [4] characterized prognostic alteration for melanoma with a panel of five genes, including *CSMD2, CNTNAP5, NRDE2, ADAM6,* and *TRPM2*. Despite considerable successes, our understanding of cancer prognosis is still limited. The limited progress in cancer analytics may be attributable to small sample sizes, high dimensionality and low signal-to-noise ratios of omics data, as well as the underlying molecular complexity of cancers.

Most of the existing studies, including the aforementioned, focus on a single type of cancer, and analysis often suffers from a lack of sufficient information. Cancer types have been typically classified according to organ- and tissue histology-based pathology criteria. This is especially true in “old” studies. More recently, with the development of high-throughput profiling, increasing attention has been paid to the molecular basis of cancers, providing a novel perspective on cancer types. A representative recent work is Hoadley, et al. [5], which conducted the molecular clustering of 33 different types of tumors in The Cancer Genome Atlas (TCGA) with data on aneuploidy, DNA methylation, mRNA, and miRNA. Their results show that some cancers, which were treated as completely different diseases according to traditional organ- and tissue histology-based pathology criteria, are closely related according to their molecular characteristics. For example, squamous cell carcinoma can occur in lung, bladder, cervix, head, and neck, and different histopathological types are often observed. However, in Hoadley, et al. [5], these cancer types have been found to have similar molecular characteristics.

Molecular similarity across cancers has been well established in the literature. Prognosis of many different cancer types is mediated by some common mechanisms associated with certain common pathways. For example, the p53 pathway inhibits cell growth and stimulates cell death, which plays an important role in a large fraction of cancers. In addition, there are other genes/pathways that have important roles in many cancer types, such as apoptosis, hypoxia-inducible transcription factor (HIF)-1, mitogen activated protein kinase (MAPK) phosphoinositide3-kinase (PI3K), and receptor tyrosine kinases (RTKs) [6]. Published studies have found that different cancer types may share common oncogenes, tumor-suppressor genes and stability genes, the alternations of which are responsible for the genesis and prognosis of cancers. For example, *BRCA1* gene mutation is often found in both breast and ovarian cancers [7]. These two cancer types are perhaps the most common cancers in female and often occur together [7]. Another example is lung adenocarcinoma and lung squamous cell carcinoma which are two major lung cancer subtypes. Many genes have been reported to be associated with both cancer subtypes, including *EGFR* [8], *TP53* [8], *AKT1*, *DDR2* [9], *FGFR1* [10], *KRAS* [8], *PTEN*, and others. With molecular similarity, one cancer may contain information useful for the analysis of other cancers. Overall, it is of interest and also reasonable to conduct the integrative analysis of molecular profiles of multiple cancer types to increase information and more accurately describe the underlying prognosis.

More recently, much effort has been devoted to collecting omics profiles of tumor samples with different cancer types under a unified protocol. A representative example is TCGA organized by The National Cancer Institute (NCI) which has generated a large amount of cross-platform genomic data for exploring the complex landscapes of human cancers. Specifically, it has collected multi-omics data from over 20,000 primary cancer and matched normal samples spanning 33 cancer types, including breast cancer, lung squamous cell carcinoma, lung adenocarcinoma, and others. Other examples include the International Cancer Genome Consortium (ICGC), Therapeutically Applicable Research to Generate Effective Treatments (TARGET), and others. With the clinical and omics data on multiple cancer types, these databases provide a good opportunity to conduct cancer modeling through data integration.

In the literature, there are a few related studies, which can be generally classified into two families. The first family adopts a meta-analysis strategy, which first analyzes different cancer types separately and then compares results across cancer types to search for overlapping findings. An example is Cava, et al. [11], which first analyzed gene expression data on 16 cancer types separately and then identified 895 de-regulated genes with a central role in pathways. Yu, et al. [12] systematically analyzed gene expressions across diverse cancers during the inflammatory timeline. After comparing the differentially expressed genes among cancers, they found three novel pan-cancer gene expression patterns, in which the gene expressions are regulated differently in the early and late phases of inflammation. Using a cohort of 3899 samples with 10 cancer types, Sharma, et al. [13] adopted a bottom-up approach to quantify the effects of gene expression variations and identified novel recurrent regulatory mutations influencing known cancer genes, such as *GRIN2D* and *NKX2-1*, in multiple cancer types. The second family of approaches stacks data from multiple cancer types together to create a “mega” dataset, and then conducts analysis as if there is in fact just a single dataset. An example is Martinez-Ledesma, et al. [14], which used a network-based exploration approach to identify gene expression biomarkers that are predictive of clinical outcomes in 12 cancer types. Using TCGA data on 3281 samples with 12 cancer types, Leiserson, et al. [15] performed a pan-cancer analysis of mutated networks with a new algorithm, HotNet2, and found some significantly mutated subnetworks as well as those with less characterized roles in cancers. Beyond studies on cancer omics data, similar strategies have also been considered in other fields of biomedical research to collectively analyze multiple datasets. For example, Xing, et al. [16] proposed two variations of a stacking algorithm to simultaneously predict the resistance of multiple drugs using mutation information, leading to improvement in prediction performance. As another example of drug analysis, Matlock, et al. [17] developed stacking models built on multiple cell lines, multiple tested drugs, as well as genomic information for drug sensitivity prediction in cancer cell lines. Medical imaging data integration has also been conducted. For example, a meta-analysis based support vector machine was introduced in [18] to collectively analyze multiple types of images, such as fluorodeoxyglucose positron emission tomography (FDG-PET) and magnetic resonance imaging (MRI), for identifying susceptible brain regions and predicting the incidence of Alzheimer’s disease.

Despite considerable successes, both families have limitations. The former neglects integration in the discovery process. Data on each cancer type still suffers from a lack of sufficient information resulting from a small sample size, high noises, and other reasons. As such, the “delay” in integration may make the analysis less effective. For the latter one, although sample size increases by stacking, subjects with different cancer types are treated as if they were from the same population. It cannot effectively accommodate the heterogeneity across cancer types. In addition, in some of the existing studies, “classic” statistical techniques have been adopted, and there is a lack of utilizing state-of-the-art techniques.

Motivated by the limitations of single cancer type analysis and recent successes of integrative analysis in other contexts, in this study our goal is to conduct more effective integrative analysis of multiple cancer types with high dimensional omics data. By contrast with the single cancer type analysis, omics data from multiple cancer types are jointly analyzed to effectively borrow information across cancer types and generate more reliable findings. By contrast with the existing meta-analysis- and stacking-based approaches, the proposed analysis integrates data on multiple cancer types in the discovery process and effectively accommodate the heterogeneity across cancer types. By contrast with the analysis on categorical and continuous outcomes, the more challenging prognosis analysis is conducted. The proposed analysis is based on the penalization technique which has a solid statistical ground and satisfactory performance in published studies. TCGA mRNA expression data on nine cancer types are analyzed to demonstrate the proposed integrative analysis approach. Overall, this study provides a practically useful new venue for cancer prognosis modeling with multiple cancer types.

## 2. Materials and Methods

### 2.1. The Cancer Genome Atlas (TCGA) Data

TCGA is one of the largest cancer genomics programs that comprehensively cover multiple cancer types with high quality omics measurements and serves as an ideal testbed. In this study, the processed level 3 data are downloaded from cBioPortal (http://www.cbioportal.org/). For omics data, we consider mRNA expressions which were measured using the IlluminaHiseq RNAseq V2 platform. For each subject, a total of 20,531 mRNA expression measurements are available. It is noted that the proposed analysis can be directly applied to other types of omics data, such as copy number variation, methylation, microRNA, and others. The prognosis outcome of interest is the overall survival time which is subject to right censoring. Nine common cancer types are analyzed, including some recognized as highly correlated, such as lung adenocarcinoma and lung squamous cell carcinoma. Summary information is provided in Table 1. We acknowledge that, as the proposed analysis can well accommodate heterogeneity across cancers, the selection of cancers for analysis does not need to follow a strict criterion. Beyond these nine cancers with high prevalence and mortality, others can be added to the analysis easily.

It has been suggested in the literature that the number of important prognostic markers is not expected to be large. Besides, with a relatively moderate sample size for each cancer type and a much larger number of genes, analysis may not be reliable. To improve estimation stability and also reduce computational cost, we conduct prescreening as follows. We consider the 1385 genes in the TruSight RNA Pan-Cancer Panel which is produced by Illunima Company and provides a comprehensive assessment of cancer-related RNA transcripts and fusion detection. These genes have been referred to in public databases and implicated in multiple cancer types, including solid tumors, soft tissue cancers, and hematological malignancies [19]. After data matching, a total of 1040 gene expression measurements are left for downstream analysis. Note that this prescreening is not essential in our analysis, and the proposed approach can be directly applied to a bigger set of genes.

### 2.2. Methods

**Figure 1:**
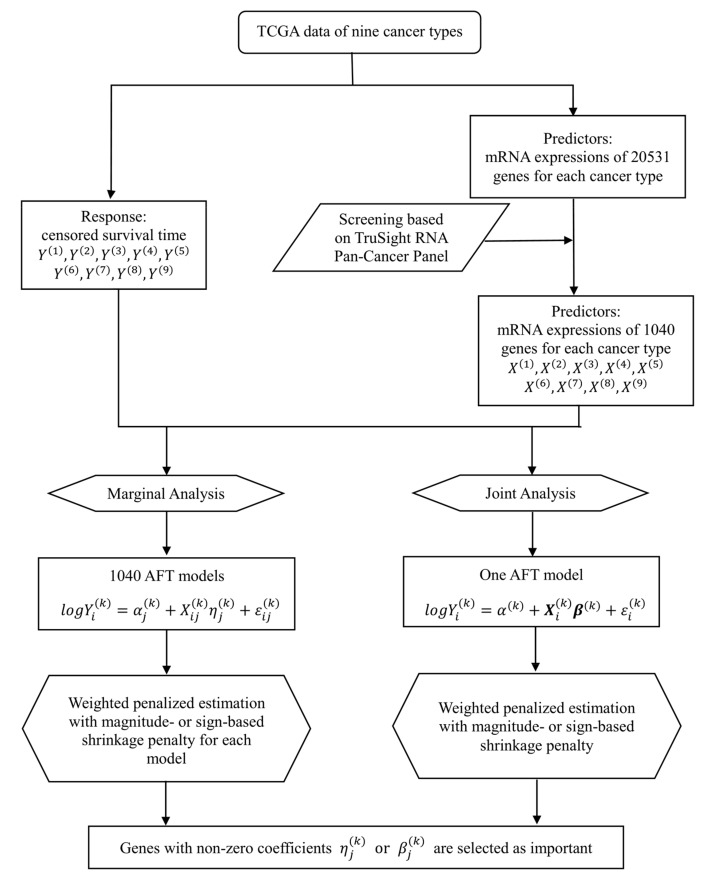
Flowchart of the proposed integrative analysis of The Cancer Genome Atlas (TCGA) data.

We conduct both marginal and joint analysis, where the former analyzes one gene at a time and the latter analyzes all genes in a single model. Both types of analysis have been extensively conducted in existing cancer modeling studies. As they have different implications and cannot replace each other, we conduct both analyses to generate a more comprehensive understanding of cancer prognosis. We develop a penalized regression-based framework to collectively analyze multiple datasets and identify markers associated with the prognosis of multiple cancer types, while effectively accounting for the similarity across cancers. The overall flowchart of analysis is provided in Figure 1.

Assume that there are K cancer types, where the $k\mathrm{th}\;\left(k=1,\ldots ,K\right)\;$ type has $n^{\left(k\right)}$ independent subjects. For subject i with the kth cancer type, let $T_{i}^{\left(k\right)}$ be the log-transformed survival time and $X_{i}^{\left(k\right)}=\left(X_{i1}^{\left(k\right)},\ldots ,X_{ip}^{\left(k\right)}\right)$ be the p-dimensional vector of gene expression measurements. In practical analysis, right censoring is usually present. Denote $C_{i}^{\left(k\right)}$ as the log-transformed censoring time, then we observe $y_{i}^{\left(k\right)}=\min\left(T_{i}^{\left(k\right)},C_{i}^{\left(k\right)}\right)$ and $\delta_{i}^{\left(k\right)}=I\left(T_{i}^{\left(k\right)}\leq C_{i}^{\left(k\right)}\right)$ with $I\left(\cdot \right)$ being the indicator function.

#### 2.2.1. Marginal Analysis

We adopt the accelerated failure time (AFT) model for describing prognosis. It has been one of the most popular choices in high-dimensional survival analysis due to its lucid interpretation and, more importantly, computational simplicity [20]. For a specific cancer type, consider the marginal AFT model for the jth measurement as:

(1) $$T_{i}^{\left(k\right)}=\alpha_{j}^{\left(k\right)}+X_{ij}^{\left(k\right)}\eta_{j}^{\left(k\right)}+\varepsilon_{ij}^{\left(k\right)},$$

where $\alpha_{j}^{\left(k\right)}$ and $\eta_{j}^{\left(k\right)}$ are the unknown intercept and coefficient, and $\varepsilon_{ij}^{\left(k\right)}$ is the random error. Assume that for each cancer type, data $\left\{\left(X_{i}^{\left(k\right)},\;y_{i}^{\left(k\right)},\delta_{i}^{\left(k\right)}\right),\;i=1,\ldots ,\;n^{\left(k\right)}\right\}$ have been sorted according to $y_{i}^{\left(k\right)}$ in an ascending order. Then, the following weighted penalized objective function is proposed to collectively analyze multiple cancer types,

(2) $$\overset{K}{\underset{k=1}{\sum }}\left[\frac{1}{2n^{\left(k\right)}}\underset{i}{\sum }w_{i}^{\left(k\right)}{\left(y_{i}^{\left(k\right)}-\alpha_{j}^{\left(k\right)}-x_{ij}^{\left(k\right)}\eta_{j}^{\left(k\right)}\right)}^{2}\right]+\overset{K}{\underset{k=1}{\sum }}\rho_{MCP}\left(\eta_{j}^{\left(k\right)},\lambda_{1},\gamma \right)+\frac{\lambda_{2}}{2}\overset{K}{\underset{k^{\prime }\neq k}{\sum }}\rho \left(\eta_{j}^{\left(k\right)},\eta_{j}^{\left(k^{\prime }\right)}\right)$$

Here, $w_{i}^{\left(k\right)}$’s are the Kaplan–Meier (KM) weights for accommodating censoring and defined as

$$w_{1}^{\left(k\right)}=\frac{\delta_{1}{}^{\left(k\right)}}{n^{\left(k\right)}},w_{i}^{\left(k\right)}=\frac{\delta_{i}{}^{\left(k\right)}}{n^{\left(k\right)}-i+1}\overset{i-1}{\underset{l=1}{\prod }}{\left(\frac{n^{\left(k\right)}-l}{n^{\left(k\right)}-l+1}\right)}^{\delta_{i}{}^{\left(k\right)}},\;\;i=2,\ldots ,n^{\left(k\right)}$$

$\rho_{MCP}\left(\left|v\right|,\lambda_{1},\gamma \right)=\lambda_{1}\int_{0}^{\left|v\right|}{\left(1-\frac{x}{\lambda_{1}\gamma }\right)}_{+}dx$ is the minimax concave penalty (MCP) with tuning parameter $\lambda_{1}$ and regularization parameter γ. We consider two types of $\rho \left(\eta_{j}^{\left(k\right)},\eta_{j}^{\left(k^{\prime }\right)}\right)$ with tuning parameter $\lambda_{2}$. The first is the magnitude-based shrinkage penalty with

(3) $$\rho \left(\eta_{j}^{\left(k\right)},\eta_{j}^{\left(k^{\prime }\right)}\right)={\left(\eta_{j}^{\left(k\right)}-s_{j}^{\left(kk^{\prime }\right)}\eta_{j}^{\left(k^{\prime }\right)}\right)}^{2},$$

where $s_{j}^{\left(kk^{\prime }\right)}=I\left(\mathrm{Sgn}\left(\eta_{j}^{\left(k\right)}\right)=\mathrm{Sgn}\left(\eta_{j}^{\left(k^{\prime }\right)}\right)\right)$ with $\mathrm{Sgn}\left(\cdot \right)$ being the sign function. The second is the sign-based shrinkage penalty with

(4) $$\rho \left(\eta_{j}^{\left(k\right)},\eta_{j}^{\left(k^{\prime }\right)}\right)={\left(\mathrm{Sgn}\left(\eta_{j}^{\left(k\right)}\right)-\mathrm{Sgn}\left(\eta_{j}^{\left(k^{\prime }\right)}\right)\right)}^{2}$$

Based on (2), a total of p objective functions are developed, and the estimates are defined as the minimizers of these objective functions. With penalization, some values of $\eta_{j}^{\left(k\right)}$’s can be shrunk to exactly zero, and variables with nonzero $\eta_{j}^{\left(k\right)}$’s are identified as important prognostic markers and associated with the kth cancer type. The magnitudes and signs of $\eta_{j}^{\left(k\right)}$’s describe the strengths and directions of associations. Following the literature, the coordinate descent (CD) technique is adopted for effectively optimizing the objective functions. Details are provided in Appendix A.

The objective function (2) analyzes one gene at a time, and enjoys stable estimation and simple optimization. It may be limited by a lack of attention to the interconnections among genes and their joint effects on cancer prognosis. Our brief literature search suggests that marginal analysis is still highly popular in high-dimensional omics studies [21]. For marginal analysis, a two-stage method is often adopted for marker identification, where multiple tests are first performed and a multiple comparison adjustment is then conducted on p values using, for example, the false discovery rate approach. By contrast with this strategy, we adopt the penalization technique, which can generate more stable results and, more importantly, effectively accommodate the similarity across cancer types. Specifically, MCP is used for regularized estimation and marker identification, which has been shown to have satisfactory theoretical and numerical properties. The most significant advancement is the $\rho \left(\eta_{j}^{\left(k\right)},\eta_{j}^{\left(k^{\prime }\right)}\right)$ penalty term which promotes similarity between the estimated coefficients of each cancer pair. Data integration is conducted in the discovery process to facilitate early information borrowing. With the magnitude-based shrinkage penalty (3), the magnitudes of gene effects across cancer types are promoted to be similar if they have the same signs, while with the sign-based shrinkage penalty (4), the signs of gene effects are promoted to be similar. Thus, the proposed two types of $\rho \left(\eta_{j}^{\left(k\right)},\eta_{j}^{\left(k^{\prime }\right)}\right)$ promote different types of similarity, with the former for *quantitative* similarity and the latter for *qualitative* similarity. As in practice the relatedness of cancer types may be not accurately known, both penalties can be useful. $\lambda_{1}$ and $\lambda_{2}$ are two tuning parameters which control the sparsity and similarity of coefficients, respectively. For the p objective functions, we impose the same values of $\lambda_{1}$ and $\lambda_{2}$ on different $\eta_{j}^{\left(k\right)}$ to be concordant with joint analysis. If $\lambda_{2}=0$, the proposed approach goes back to the unintegrated strategy that analyzes each cancer type separately with MCP.

#### 2.2.2. Joint Analysis

For k=1,…,K, consider the AFT model with the joint effects of all omics measurements,

(5) $$\;T_{i}^{\left(k\right)}=\alpha^{\left(k\right)}+X_{i}^{\left(k\right)}\beta^{\left(k\right)}+\varepsilon_{i}^{\left(k\right)},$$

where $\alpha^{\left(k\right)}$ is the intercept, $\beta^{\left(k\right)}={\left(\beta_{1}^{\left(k\right)},\ldots ,\beta_{p}^{\left(k\right)}\right)}^{\prime }$ is the p-dimensional unknown coefficient vector, and $\varepsilon_{i}^{\left(k\right)}$ is the random error. With the same notations as in the marginal analysis, for estimation, consider the following weighted penalized objective function

(6) $$\overset{K}{\underset{k=1}{\sum }}\left[\frac{1}{2n^{\left(k\right)}}\underset{i}{\sum }w_{i}^{\left(k\right)}{\left(y_{i}^{\left(k\right)}-\alpha^{\left(k\right)}-X_{i}^{\left(k\right)}\beta^{\left(k\right)}\right)}^{2}\right]+\overset{K}{\underset{k=1}{\sum }}\overset{p}{\underset{j=1}{\sum }}\rho_{MCP}\left(\beta_{j}^{\left(k\right)},\lambda_{3},\gamma \right)+\frac{\lambda_{4}}{2}\underset{k^{\prime }\neq k}{\sum }\overset{p}{\underset{j=1}{\sum }}\rho \left(\beta_{j}^{\left(k\right)},\beta_{j}^{\left(k^{\prime }\right)}\right),$$

where $\lambda_{3}$ and $\lambda_{4}$ are the tuning parameters. The KM weights, MCP, and two proposals for $\rho \left(\beta_{j}^{\left(k\right)},\beta_{j}^{\left(k^{\prime }\right)}\right)$ are also adopted in (6). The proposed estimate is defined as the minimizer of (6). Variables with nonzero estimates are identified as associated with prognosis. For optimization, the CD algorithm is adopted (Appendix A).

Different from (2), objective function (6) jointly analyzes a large number of genes in a single model and thus accommodates a high dimensionality. Compared to marginal analysis, it advances by taking the combined effects of multiple genes into consideration and better describing the underlying disease biology. However, it involves more complex computation and may lead to less stable results. Penalization is adopted to accommodate high dimensionality and identify important genes. It is perhaps the most popular technique in high dimensional data analysis. Different from the existing studies, the magnitude- and sign-based shrinkage penalty terms are also introduced similarly to that in Section 2.2.1. This can effectively accommodate the similarity across cancer types and facilitate information borrowing.

The proposed analysis can be effectively realized. To facilitate data analysis within and beyond this study, we have developed R code and made it publicly available at www.github.com/shuanggema/IntePanCancer.

## 3. Results

### 3.1. Marginal Analysis

We analyze the TCGA data using the approach described in Section 2.2.1 with penalties (3) (referred to as A1) and (4) (referred to as A2), as well as an alternative marginal approach A3 which analyzes each cancer type separately with MCP for identifying relevant markers. Comparing with the benchmark A3 can straightforwardly establish the merit of the proposed integrative analysis. Detailed estimation results are provided in the Supplementary Excel file. Different approaches are observed to generate different findings. Specifically, a total of 910 genes with 482 unique ones and 1160 genes with 275 unique ones are identified with A1 and A2, respectively, compared to 2655 genes with 999 unique ones with A3.

In Table 2, we present the top five genes with the largest numbers of associated cancer types and refer to the Supplementary Excel file for more detailed results. It is observed that the numbers of multiple cancer types-related genes identified with A1 and A2 are slightly larger than those with A3. For example, both A1 and A2 identify gene *APH1A* as associated with all nine cancer types, but this gene is missed by A3. Literature search suggests that the identified genes with the proposed A1 and A2 may have important biological implications. For example, the Kyoto Encyclopedia of Genes and Genomes (KEGG) pathway analysis of gene *APH1A* suggests that it is a member of the notch signaling pathway which has an important impact on developmental and cell fate decisions and is deregulated in human solid tumors [22]. APH1A is one of the four essential components of γ-secretase [23]. γ-secretase is a multiprotein intramembrane-cleaving protease, which can cleave ligand-activated endogenous Notch receptors and is a potential drug target for cancer [24]. Gene *MAPK1*, identified as associated with eight cancer types by both A1 and A2, has been reported to be involved in many cancer related pathways. MAPK1 is one of the MAP kinases in the MAPK pathway. It can phosphorylate transcription factors, which regulate the expressions of genes involved in cell proliferation and differentiation. Besides, MAPK1 is involved in EGFR tyrosine kinase inhibitor resistance [25], which importantly contributes to the etiology of various types of cancer, such as pancreatic cancer [26], paediatric acute lymphoblastic leukemia [27], and others. In this process, MAPK1 acts as a serine/threonine kinase upstream of FRS2, which plays a role in epidermal growth factor (EGF) signaling [28]. MAPK1 has also been reported to have an impact on the malignant behavior of breast cancer cells. Published studies show that gene *ETV6*, identified as associated with eight and seven cancer types by A1 and A2, respectively, is involved in the transcriptional dysregulation of cancer pathways. The dysregulation of transcription factors can alter the expressions of target genes and lead to the tumorigenic process. For example, ETV6 is a negative regulator of transcription 3 (Stat3) transcription factor activity, which has the ability to mediate the inhabitation of the proliferation of tumor cells [29]. Gene *ETV6* is relevant to multiple cancer types, including breast cancer [30], leukemia [31], non-small cell lung cancer [32], and others. These biological findings provide support to the validity of the proposed integrative analysis.

To gain a deeper insight into the identification results, we further calculate the relative overlapping between gene sets associated with different cancer types. Specifically, for two gene sets A and B, their relative overlapping is defined as $\mathrm{ROL}\left(A,B\right)=\frac{A\cap B}{A\cup B}$, with a larger value indicating a stronger similarity. Results for different approaches are shown in Table 3. The average ROL values are 0.143 (A1), 0.308 (A2), and 0.147 (A3), respectively, suggesting that A2 leads to gene sets with a higher level of relative overlapping and A1 and A3 have comparable performance. Take breast invasive carcinoma (BRCA) and ovarian serous cystadenocarcinoma (OV), which are established as related, as an example. The ROL values for A1, A2, and A3 are 0.150, 0.265, and 0.146, respectively. The proposed A2 can improve the *qualitative* similarity of genes selected for multiple cancer types to a certain extent.

**Figure A1:**
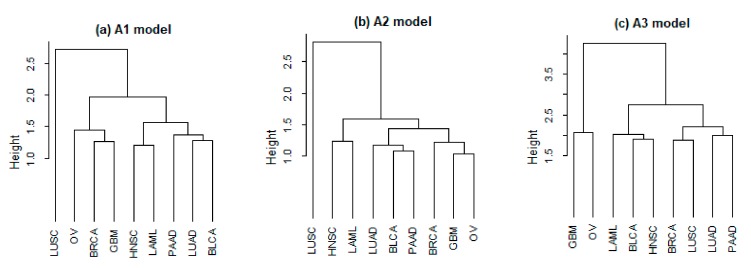
Marginal analysis: clustering dendrogram based on the relative Euclidean distances.

Beyond identification, we also take a closer look at the estimation results. Specifically, we compute the difference of the estimated coefficient matrices for each cancer pair. Consider the relative Euclidean distance defined as $\;\overset{p}{\underset{j=1}{\sum }}{\left(\eta_{j}^{\left(k\right)}-\eta_{j}^{\left(k^{\prime }\right)}\right)}^{2}/\sqrt{\overset{p}{\underset{j=1}{\sum }}{\left(\eta_{j}^{\left(k\right)}\right)}^{2}\overset{p}{\underset{j=1}{\sum }}{\left(\eta_{j}^{\left(k^{\prime }\right)}\right)}^{2}}$ for $k\neq k^{\prime }$, with a smaller value indicating a stronger similarity. Results for the three approaches are provided in Table 4, with the average values being 1.606 (A1), 1.534 (A2), and 2.254 (A3). The relative Euclidean distances with A1 and A2 are observed to be smaller than those with A3. For example, the distance values between BRCA and OV are 1.443 with A1 and 1.220 with A2, which are much smaller than 3.230 with A3. As another example, for the two squamous cell carcinomas, lung squamous cell carcinoma (LUSC) and head and neck squamous cell carcinoma (HNSC), the relative Euclidean distances are 1.644 (A1), 1.855 (A2), and 2.577 (A3), respectively. To more intuitively describe similarity, we conduct the hierarchical clustering analysis based on the relative Euclidean distances and present the results in Figure A1 (Appendix B). Biologically sensible findings are made, for example, the distance between BRCA and OV decreases after integration.

### 3.2. Joint Analysis

Similar to marginal analysis, in joint analysis we adopt both the magnitude-based shrinkage (referred to as B1) and the sign-based shrinkage (referred to as B2). We also consider an alternative joint analysis referred to as B3, which analyzes each cancer type separately and applies MCP to accommodate high dimensionality and select relevant markers. Detailed estimation results are provided in the Supplementary Excel file. For the nine cancer types combined, B1, B2, and B3 identify a total of 1135 genes with 662 unique ones, 1064 genes with 598 unique ones, and 530 genes with 421 unique ones, respectively. The two proposed approaches lead to results different from the alternative. In addition, the joint analysis identification results also differ from those in marginal analysis.

The top five genes with the largest numbers of associated cancer types are provided in Table 5, and more results are provided in the Supplementary Excel file. Similar patterns are observed where the proposed two approaches identify more genes associated with multiple cancer types. For the identified genes, a literature search provides independent evidences of their associations with multiple cancer types. For example, the important biological implications of gene *APH1A* have been already discussed in Section 3.1. In addition, gene *CCAR2*, identified as important for all nine cancer types with B2, has been reported to be associated with the development of many cancer types. It plays a pivotal role in DNA damage response and promoting apoptosis. The depletion of *CCAR2* can impair the activation of the AKT pathway, which ultimately causes the inhibition of cancer cell growth [33]. Specifically, it binds to the BRCA1 C Terminus (BRCT) domain of the tumor suppressor BRCA1 and inhibits BRCA1 in breast cancer [34]. Cho, et al. [35] also suggested that the expression of *CCAR2* is closely related with the progression of ovarian carcinomas. In Kim, et al. [36], an increase in apoptosis was observed in *CCAR2*-deficient non-small cell lung cancer cell lines. Wagle, et al. [37] demonstrated that the expression of *CCAR2* is significantly associated with a higher clinical stage and predicted shorter survival in osteosarcoma. Gene *BTLA* is identified as important for eight cancer types with B2. It is an immunoinhibitory receptor and can deliver inhibitory signals for suppressing lymphocyte activation. The ability of *BTLA* to inhibit tumor-specific human CD8+ T cells suggests it as a target for cancer immunotherapy [38]. Published studies also suggest that gene *BTLA* is relevant to the occurrence and development of many cancer types [39]. For example, a case-control study conducted by Fu, et al. [40] on women from northeast China suggested that breast cancer risk and prognosis may be affected by *BTLA* gene polymorphisms. In addition, Oguro, et al. [41] showed that *BTLA* is closely associated with shorter overall survival in gallbladder cancer. Gene *RUNX2* is identified by B2 as important for five cancer types. The transcription factor RUNX2 can regulate the expressions of genes that are associated with tumor promotion, invasion, and metastasis, such as *VEGF* [42]. *RUNX2* is also involved in many pathways that are related to tumorigenesis, such as the WNT pathway, transforming growth factor beta (TGFβ) signaling pathway, and p53 pathway [42].

**Figure A2:**
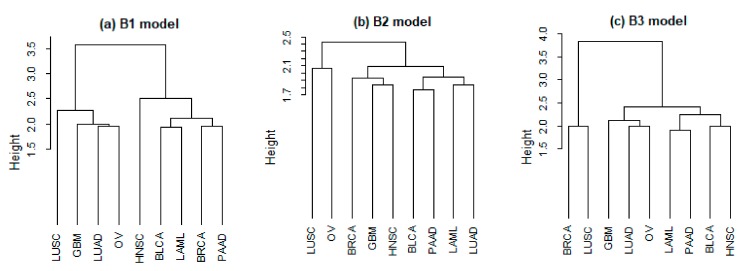
Joint analysis: clustering dendrogram based on the relative Euclidean distances.

The relative overlapping and Euclidean distances between different cancer types are presented in Table A1 and Table A2 (Appendix B). The average values of relative overlapping are 0.103 (B1), 0.107 (B2), and 0.030 (B3), and the average values of Euclidean distance are 2.261 (B1), 1.980 (B2), and 2.459 (B3). Both measures indicate that the proposed joint integrative analysis can improve the identified similarity across cancer types. Take BRCA and PAAD, the relatedness of which has been suggested in literature, as an example. It has been demonstrated that protein annexin A1, A2, A4 and A5 play an important role in the occurrence and development of these two cancer types [43], and *BRCA1* and *BRCA2* gene mutations are commonly observed in both cancer types [44]. The values of relative overlapping are 0.074 (B1), 0.116 (B2), and 0.027 (B3), and the relative Euclidean distances are 1.949 (B1), 1.906 (B2), and 3.829 (B3). For the two common lung cancer subtypes, lung adenocarcinoma (LUAD) and LUSC, the relative overlapping values are 0.098 (B1), 0.119 (B2), and 0.039 (B3), and the relative Euclidean distances are 2.250 (B1), 2.012 (B2), and 2.998 (B3). Results of hierarchical clustering analysis based on the relative Euclidean distances are shown in Figure A2 (Appendix B). With the proposed B1 and B2, cancer types with stronger relatedness tend to be assigned to the same clusters.

Advancing from marginal analysis, joint analysis has the capability of predicting survival time besides marker identification. To evaluate prediction performance, a resampling procedure is adopted. Specifically, for each of the nine cancers, we first split data randomly into a training and a testing set. The training sets for the nine cancer types are then used to fit models and obtain parameter estimates. Finally, we make prediction for the testing set subjects with the estimated parameters. For evaluation, C-statistic is adopted, which is one of the most popular measures for censored survival data [45,46]. It is the integrated AUC (area under the curve) of the time-dependent ROC curve and has value between 0.5 and 1, with a larger value indicating a better prediction performance. The average values over 100 resamplings are shown in Table 6. Overall, B1 and B2 perform better than B3, with B1 having a prominent superiority. For example, for LUSC, the average C-statistic values are 0.748 (B1), 0.649 (B2), and 0.612 (B3). The improvement in prediction accuracy suggests the benefit of integrative analysis of multiple cancer types.

### 3.3. Simulation Based on TCGA Data

To gain more insights into the performance of the proposed integrative analysis, we conduct practical data-based simulation under various scenarios. The specific settings were as follows. (1) The observed gene expression measurements on nine cancer types from TCGA were used as predictors. To generate variations across simulation replicates, we adopted a resampling approach. (2) Set p=200, 500, or 1000. For each value of p, genes were randomly selected from the original gene set. (3) For each cancer type, there were 10 genes associated with the cancer outcomes with nonzero regression coefficients $\beta_{\left(1\right)}^{\left(k\right)},\ldots ,\;\beta_{\left(10\right)}^{\left(k\right)}$. The rest of the coefficients were zeros. (4) For each subject, the event time was computed from the AFT model $\log\left(T_{i}^{\left(k\right)}\right)=\overset{5}{\underset{j=1}{\sum }}x_{i\left(j\right)}^{\left(k\right)}\beta_{\left(j\right)}^{\left(k\right)}+\overset{10}{\underset{j=6}{\sum }}{\left(x_{i\left(j\right)}^{\left(k\right)}\right)}^{2}\beta_{\left(j\right)}^{\left(k\right)}+\varepsilon_{i}$, where the random error $\varepsilon_{i}$ was generated from $N\left(0,1\right)$. Censoring times were randomly generated from an exponential distribution, and the parameter was adjusted to make the censoring rate around 20%. It is noted that to mimic the complexity of real data, the data generating models are more complicated than the simple AFTs with the presence of a small number of quadratic effects. We consider various values of $\beta_{\left(1\right)}^{\left(k\right)},\ldots ,\;\beta_{\left(10\right)}^{\left(k\right)}$ to generate different levels of signal-to-noise ratios and cancer similarity. Under Scenarios I and II, the nine cancer types have the same set of important genes with the same nonzero effects. In particular, for j=1,…, 10 and k=1,…,9, we set $\beta_{\left(j\right)}^{\left(k\right)}=5$ and 2 for Scenarios I and II, respectively. Under Scenario III, the nine cancer types have the same set of important genes, but the magnitudes of effects vary. Specifically, $\beta_{\left(j\right)}^{\left(k\right)}$’s are randomly generated from $U\left(1,5\right)$. Under Scenario IV, the nine cancer types have different sets of important genes. Specifically, the first five important genes have the same effects for all nine cancer types with $\beta_{\left(j\right)}^{\left(k\right)}=2$, and the other five important genes are “randomly selected” (and hence likely to differ across datasets) and with $\beta_{\left(j\right)}^{\left(k\right)}=2$. There are a total of 12 simulation settings, comprehensively covering different numbers of genes, and different levels of signal-to-noise ratios and cancer similarity.

Analysis was conducted using the proposed marginal and joint analysis approaches as well as two alternatives. To evaluate identification performance, we computed the true positive rate (TPR) and false positive rate (FPR). The average TPR and FPR values over 100 replicates are provided in Table A3, together with the numbers of the identified true positives associated with all nine cancer types (NG). Overall, the four integrative analysis approaches perform better than the two alternatives, with larger values of TPR and smaller values of FPR. For example, under Scenario I with p=200, the average values of (TPR, FPR) are (0.980, 0.258) with A1, (0.951, 0.185) with A2, (0.944, 0.641) with A3, (0.838, 0.087) with B1, (0.880, 0.085) with B2, and (0.688, 0.200) with B3, respectively. The proposed approaches also identify genes with more overlaps across cancer types. Under this specific setting, the average values of NG are 7.0 (A1), 8.4 (A2), 3.8 (A3), 5.7 (B1), 8.8 (B2), and 1.4 (B3). Compared to Scenario I which has a higher signal-to-noise ratio, performance of all six approaches decay under Scenarios II–IV. Similar patterns are observed when dimensionality increases, where all approaches behave worse. However, the proposed approaches still have favorable performance. Take Scenario IV with p=500 as an example, the proposed A1, A2, B1, and B2 have (TPR, FPR) = (0.822, 0.058), (0.678, 0.054), (0.864, 0.040), and (0.719, 0.046), compared to (0.617, 0.116) with A3 and (0.646, 0.038) with B3. In addition, the average values of NG are 4.6 (A1), 2.6 (A2), 0.0 (A3), 5.0 (B1), 3.2 (B2), and 1.8 (B3). As the sign consistency of some genes does not hold under Scenario IV, A2 and B2 have inferior performance compared to A1 and B1, but still have superior performance compared to A3 and B3. The superiority of the proposed integrative analysis approaches observed in data-based simulation provides certain confidence to data analysis results.

## 4. Discussion

In cancer research, prognosis modeling with omics measurements plays an essential role. The existing studies mostly conduct analysis on one single type of cancer and often suffer from a lack of sufficient information. Integrative analysis represents an emerging trend in recent biomedical studies, among which the most common is the integrative analysis of multiple types of omics data, including gene expressions, copy number variations, and some others, and has led to interesting findings beyond single type omics data-based analysis. In this study, we have taken a different perspective and conducted integrative analysis on multiple cancer types to facilitate across-cancer information borrowing. Similarity across cancer types has been extensively studied in the literature, which provides a solid biological ground for our integrative analysis. Both marginal and joint analysis have been developed with two types of similarity-based penalty, which have intuitive formulations and solid statistical basis. We have analyzed mRNA gene expression data on nine TCGA cancer types with censored survival outcomes. Biologically sensible findings different from the benchmark analysis have been made.

The proposed analysis can be directly applied to other types of omics data and other cancer types. In this study, we have focused on prognosis data and the AFT model. A continuous outcome can be regarded as a special case of prognosis outcome without censoring, and thus the proposed analysis can be applied directly. It can also be extended to accommodate categorical outcomes using, for example, generalized linear models. With the availability of multiple types of omics data on multiple cancer types, it can be of interest to conduct the two types of integration simultaneously. More functional examination of the data analysis results will be needed to confirm the findings.

## Figures and Tables

**Table 1:** Summary information of the nine cancer types.

Cancer Type	Abbreviation	Sample Size	Non-Censored	Overall Survival (Month)	Median Survival
Breast invasive carcinoma	BRCA	802	119	0.03–282.69	29.88
Bladder Urothelial Carcinoma	BLCA	409	180	0.43–165.90	17.61
Glioblastoma multiforme	GBM	541	417	0.10–127.60	10.70
Head and Neck squamous cell carcinoma	HNSC	159	69	0.07–135.19	12.48
Acute Myeloid Leukemia	LAML	199	132	0.10–118.10	17.00
Lung adenocarcinoma	LUAD	509	183	0.13–238.11	21.62
Lung squamous cell carcinoma	LUSC	497	215	0.03–173.69	21.91
Ovarian serous cystadenocarcinoma	OV	582	384	0.26–180.06	33.03
Pancreatic adenocarcinoma	PAAD	184	100	0.13–90.05	15.34

**Table 2:** Marginal analysis: top five genes with the largest numbers of associated cancer types.

Approach	Gene	Number of Associated Cancer Types
A1	*APH1A*	9
	*ETV6*	8
	*MAPK1*	8
	*MDS2*	8
	*AKT2*	7
A2	*APH1A*	9
	*CXCR4*	8
	*MAPK1*	8
	*ACVR2A*	7
	*ETV6*	7
A3	*LAMA1*	8
	*IGF1*	7
	*NAPA*	7
	*TCTA*	7
	*TNFRSF10D*	7

**Table 3:** Marginal analysis: relative overlapping between different cancer types.

Approach		BRCA	GBM	HNSC	LAML	LUAD	LUSC	OV	PAAD
A1	BLCA	0.134	0.145	0.135	0.147	0.189	0.070	0.145	0.191
	BRCA		0.167	0.120	0.145	0.148	0.072	0.150	0.119
	GBM			0.149	0.169	0.215	0.089	0.208	0.119
	HNSC				0.202	0.199	0.108	0.154	0.160
	LAML					0.144	0.140	0.134	0.165
	LUAD						0.102	0.141	0.173
	LUSC							0.114	0.058
	OV								0.117
A2	BLCA	0.314	0.267	0.320	0.295	0.351	0.161	0.185	0.337
	BRCA		0.272	0.330	0.306	0.234	0.262	0.265	0.341
	GBM			0.416	0.443	0.271	0.310	0.364	0.237
	HNSC				0.346	0.380	0.253	0.366	0.430
	LAML					0.337	0.398	0.302	0.338
	LUAD						0.241	0.298	0.339
	LUSC							0.292	0.201
	OV								0.286
A3	BLCA	0.252	0.082	0.168	0.102	0.196	0.252	0.124	0.162
	BRCA		0.091	0.245	0.101	0.255	0.364	0.146	0.191
	GBM			0.052	0.055	0.065	0.069	0.060	0.071
	HNSC				0.116	0.198	0.251	0.096	0.162
	LAML					0.105	0.108	0.135	0.074
	LUAD						0.227	0.115	0.176
	LUSC							0.134	0.197
	OV								0.081

**Table 4:** Marginal analysis: relative Euclidean distances between estimated coefficient matrices.

Approach		BRCA	GBM	HNSC	LAML	LUAD	LUSC	OV	PAAD
A1	BLCA	1.426	1.465	1.572	1.422	1.277	2.160	1.441	1.318
	BRCA		1.270	1.853	1.551	1.457	2.445	1.443	1.584
	GBM			1.974	1.658	1.389	2.722	1.362	1.766
	HNSC				1.205	1.424	1.644	1.530	1.382
	LAML					1.403	1.591	1.471	1.373
	LUAD						1.960	1.463	1.376
	LUSC							1.942	1.959
	OV								1.532
A2	BLCA	1.166	1.250	1.435	1.378	1.075	2.814	1.424	1.070
	BRCA		1.202	1.585	1.482	1.356	2.800	1.220	1.111
	GBM			1.384	1.176	1.293	2.746	1.028	1.307
	HNSC				1.236	1.288	1.855	1.465	1.118
	LAML					1.269	1.764	1.457	1.252
	LUAD						2.347	1.337	1.160
	LUSC							2.512	2.658
	OV								1.205
A3	BLCA	2.354	2.162	1.896	2.029	2.217	2.752	2.203	2.099
	BRCA		2.862	2.364	2.514	1.974	1.870	3.230	1.956
	GBM			2.108	1.929	2.832	2.731	1.835	2.613
	HNSC				1.985	2.151	2.577	2.237	1.916
	LAML					2.455	2.405	2.221	2.207
	LUAD						2.019	3.100	1.988
	LUSC							3.154	2.206
	OV								2.871

**Table 5:** Joint analysis: top five genes with the largest numbers of associated cancer types.

Approach	Gene	Number of Associated Cancer Types
B1	ETV6	6
	GOT1	6
	CHIC2	5
	CSNK2A1	5
	RUNX2	5
B2	APH1A	9
	CCAR2	9
	HIST1H2AL	9
	BTLA	8
	LAMA1	8
B3	EPO	4
	FASLG	4
	WDR18	4
	CCND2	3
	CRADD	3

**Table 6:** Joint analysis: prediction performance of different approaches (mean C-statistic).

	BLCA	BRCA	GBM	HNSC	LAML	LUAD	LUSC	OV	PAAD
B1	0.665	0.876	0.604	0.641	0.573	0.688	0.748	0.577	0.689
B2	0.597	0.719	0.581	0.567	0.551	0.601	0.649	0.562	0.632
B3	0.587	0.693	0.558	0.604	0.558	0.594	0.612	0.547	0.589

## Supplementary Materials

The following are available online at https://www.mdpi.com/2073-4425/10/8/604/s1: Detailed results referred to in Section 3 are available in the Supplementary Excel file. Table S1: Detailed estimation and identification results.

## Appendix A

For optimizing objective functions (2) and (6), a weighted normalization is first conducted as:

$$y_{i}^{\left(k\right)}=\sqrt{w_{i}^{\left(k\right)}}\left(y_{i}^{\left(k\right)}-{\bar{y}}^{\left(k\right)}\right),\;\;x_{ij}^{\left(k\right)}=\sqrt{w_{i}^{\left(k\right)}}\left(x_{ij}^{\left(k\right)}-{\bar{x_{j}}}^{\left(k\right)}\right),$$

where ${\bar{y}}^{\left(k\right)}=\overset{n^{\left(k\right)}}{\underset{i=1}{\sum }}w_{i}^{\left(k\right)}y_{i}^{\left(k\right)}/\overset{n^{\left(k\right)}}{\underset{i=1}{\sum }}w_{i}^{\left(k\right)}$ and ${\bar{x_{j}}}^{\left(k\right)}=\overset{n^{\left(k\right)}}{\underset{i=1}{\sum }}w_{i}^{\left(k\right)}x_{ij}^{\left(k\right)}/\overset{n^{\left(k\right)}}{\underset{i=1}{\sum }}w_{i}^{\left(k\right)}$. Then objective functions (2) and (6) can be rewritten as:

(A1) $$\sum_{k=1}^{K}\left[\frac{1}{2n^{\left(k\right)}}\sum_{i}{\left(y_{i}^{\left(k\right)}-x_{ij}^{\left(k\right)}\eta_{j}^{\left(k\right)}\right)}^{2}\right]+\sum_{k=1}^{K}\rho_{MCP}\left(\eta_{j}^{\left(k\right)},\lambda_{1},\gamma \right)+\frac{\lambda_{2}}{2}\sum_{k^{\prime }\neq k}^{K}\rho \left(\eta_{j}^{\left(k\right)},\eta_{j}^{\left(k^{\prime }\right)}\right),$$

and

(A2) $$\sum_{k=1}^{K}\left[\frac{1}{2n^{\left(k\right)}}\sum_{i}{\left(y_{i}^{\left(k\right)}-X_{i}^{\left(k\right)}\beta^{\left(k\right)}\right)}^{2}\right]+\sum_{k=1}^{K}\sum_{j=1}^{p}\rho_{MCP}\left(\beta_{j}^{\left(k\right)},\lambda_{3},\gamma \right)+\frac{\lambda_{4}}{2}\sum_{k^{\prime }\neq k}\sum_{j=1}^{p}\rho \left(\beta_{j}^{\left(k\right)},\beta_{j}^{\left(k^{\prime }\right)}\right).\;$$

The coordinate descent (CD) technique is used to optimize objective functions (A1) and (A2). In the CD procedure, the objective function is optimized with respect to one parameter at a time, and the other parameters are fixed at their current values. All parameters are iteratively cycled through until convergence.

Specifically, with fixed tuning parameters, for j=1,…,p,  the CD algorithm for penalized objective function (A1) proceeds as follows.

(1). Initialize t=0, ${\left(\eta_{j}^{\left(k\right)}\right)}^{\left(t\right)}=0,\;k=1,\;..,K,$ where ${\left(\eta_{j}^{\left(k\right)}\right)}^{\left(t\right)}$ denotes the estimate of $\eta_{j}^{\left(k\right)}$ at iteration t.
(2). For k=1, …,K, carry out the following steps sequentially.
  (2.1) If $\;\rho \left(\eta_{j}^{\left(k\right)},\eta_{j}^{\left(k^{\prime }\right)}\right)$ is the magnitude-based shrinkage penalty (3), compute:
    $$b=\frac{1}{n^{\left(k\right)}}\sum_{i=1}^{n^{\left(k\right)}}x_{ij}^{\left(k\right)}{}^{2}+\lambda_{2}\;\mathrm{and}\;a=\frac{1}{n^{\left(k\right)}}\sum_{i=1}^{n^{\left(k\right)}}x_{ij}^{\left(k\right)}y_{i}^{\left(k\right)}+\lambda_{2}\sum_{k^{\prime }\neq k}s_{j}^{\left(kk^{\prime }\right)}{\left(\eta_{j}^{\left(k^{\prime }\right)}\right)}^{\left(t\right)}.$$
    If $\rho \left(\eta_{j}^{\left(k\right)},\eta_{j}^{\left(k^{\prime }\right)}\right)$ is the sign-based shrinkage penalty (4), compute:
    $$b=\frac{1}{n^{\left(k\right)}}\sum_{i=1}^{n^{\left(k\right)}}x_{ij}^{{\left(k\right)}^{2}}+\frac{\lambda_{2}}{{\left({\left(\eta_{j}^{\left(k\right)}\right)}^{\left(t\right)}+\chi \right)}^{2}},\;a=\frac{1}{n^{\left(k\right)}}\sum_{i=1}^{n^{\left(k\right)}}x_{ij}^{\left(k\right)}y_{i}^{\left(k\right)}+\lambda_{2}\sum_{k^{\prime }\neq k}\frac{{\left(\eta_{j}^{\left(k^{\prime }\right)}\right)}^{\left(t\right)}}{\left({\left(\eta_{j}^{\left(k\right)}\right)}^{\left(t\right)}+\chi \right)\left({\left(\eta_{j}^{\left(k^{\prime }\right)}\right)}^{\left(t\right)}+\chi \right)},$$
    where χ is a small positive number, which is set as 0.01 in our numerical study.
  (2.2) If $\left|\frac{a}{b}\right|>\gamma \lambda_{1}$, update ${\left(\eta_{j}^{\left(k\right)}\right)}^{\left(t+1\right)}=\frac{a}{b}$;
    else if $\left|a\right|>\lambda_{1}$, update ${\left(\eta_{j}^{\left(k\right)}\right)}^{\left(t+1\right)}=\frac{a-Sgn\left(a\right)*\lambda_{1}}{\left(b-1\right)/\gamma }$;
    else, update ${\left(\eta_{j}^{\left(k\right)}\right)}^{\left(t+1\right)}=0$.
(3). Repeat Step (2) until convergence. In our numerical study, convergence is concluded if $\sum_{k=1}^{K}\left|{\left(\eta_{j}^{\left(k\right)}\right)}^{\left(t+1\right)}-{\left(\eta_{j}^{\left(k\right)}\right)}^{\left(t\right)}\right|<10^{-4}$.

With fixed tuning parameters, the CD algorithm for penalized objective function (A2) proceeds as follows.

(1). Initialize t=0, ${\left(\beta^{\left(k\right)}\right)}^{\left(t\right)}={\left(0,\ldots ,0\right)}^{\prime },\;k=1,\;..,K,$ where ${\left(\beta^{\left(k\right)}\right)}^{\left(t\right)}$ denotes the estimate of $\beta^{\left(k\right)}$ at iteration t.
(2). For j=1, …,p and  k=1, …,K, carry out the following steps sequentially.
  (2.1) If $\;\rho \left(\beta_{j}^{\left(k\right)},\beta_{j}^{\left(k^{\prime }\right)}\right)$ is the magnitude-based shrinkage penalty (3), compute:
    $$b=\frac{1}{n^{\left(k\right)}}\overset{n^{\left(k\right)}}{\underset{i=1}{\sum }}x_{ij}^{\left(k\right)}{}^{2}+\lambda_{4},\;\mathrm{and}\;a=\frac{1}{n^{\left(k\right)}}\overset{n^{\left(k\right)}}{\underset{i=1}{\sum }}x_{ij}^{\left(k\right)}\left(y_{i}^{\left(k\right)}-\overset{p}{\underset{j^{\prime }\neq j}{\sum }}x_{ij^{\prime }}^{\left(k\right)}\beta_{j^{\prime }}^{\left(k\right)}\right)+\lambda_{2}\underset{k^{\prime }\neq k}{\sum }s_{j}^{\left(kk^{\prime }\right)}{\left(\beta_{j}^{\left(k^{\prime }\right)}\right)}^{\left(t\right)}.$$
    If $\rho \left(\beta_{j}^{\left(k\right)},\beta_{j}^{\left(k^{\prime }\right)}\right)$ is the sign-based shrinkage penalty (4), compute:
    $$b=\frac{1}{n^{\left(k\right)}}\sum_{i=1}^{n^{\left(k\right)}}x_{ij}^{{\left(k\right)}^{2}}+\frac{\lambda_{4}}{{\left({\left(\beta_{j}^{\left(k\right)}\right)}^{\left(t\right)}+\chi \right)}^{2}},\;a=\frac{1}{n^{\left(k\right)}}\sum_{i=1}^{n^{\left(k\right)}}x_{ij}^{\left(k\right)}\left(y_{i}^{\left(k\right)}-\sum_{j^{\prime }\neq j}^{p}x_{ij^{\prime }}^{\left(k\right)}\beta_{j^{\prime }}^{\left(k\right)}\right)+\lambda_{2}\underset{k^{\prime }\neq k}{\sum }\frac{{\left(\beta_{j}^{\left(k^{\prime }\right)}\right)}^{\left(t\right)}}{\left({\left(\beta_{j}^{\left(k\right)}\right)}^{\left(t\right)}+\chi \right)\left({\left(\beta_{j}^{\left(k^{\prime }\right)}\right)}^{\left(t\right)}+\chi \right)},$$
    where χ is a small positive number, which is set as 0.01 in our numerical study.
  (2.2) If $\left|\frac{a}{b}\right|>\gamma \lambda_{3}$, update ${\left(\beta_{j}^{\left(k\right)}\right)}^{\left(t+1\right)}=\frac{a}{b}$;
    else if $\left|a\right|>\lambda_{3}$, update ${\left(\beta_{j}^{\left(k\right)}\right)}^{\left(t+1\right)}=\frac{a-Sgn\left(a\right)*\lambda_{3}}{\left(b-1\right)/\gamma }$;
    else, update ${\left(\beta_{j}^{\left(k\right)}\right)}^{\left(t+1\right)}=0$.
(3). Repeat Step (2) until convergence. In our numerical study, convergence is concluded if $\overset{p}{\underset{j=1}{\sum }}\overset{K}{\underset{k=1}{\sum }}\left|{\left(\beta_{j}^{\left(k\right)}\right)}^{\left(t+1\right)}-{\left(\beta_{j}^{\left(k\right)}\right)}^{\left(t\right)}\right|<10^{-4}$.

These approaches involve tuning parameters, which are selected using cross validation.

## Appendix B

**Table A1:** Joint analysis: relative overlapping between different cancer types.

Approach		BRCA	GBM	HNSC	LAML	LUAD	LUSC	OV	PAAD
B1	BLCA	0.075	0.087	0.114	0.124	0.116	0.126	0.087	0.105
	BRCA		0.068	0.069	0.152	0.086	0.082	0.099	0.074
	GBM			0.138	0.129	0.085	0.106	0.089	0.114
	HNSC				0.114	0.121	0.086	0.112	0.071
	LAML					0.137	0.11	0.129	0.088
	LUAD						0.098	0.114	0.083
	LUSC							0.127	0.097
	OV								0.091
B2	BLCA	0.097	0.085	0.090	0.128	0.124	0.121	0.124	0.107
	BRCA		0.095	0.088	0.104	0.091	0.102	0.133	0.116
	GBM			0.101	0.113	0.071	0.109	0.124	0.127
	HNSC				0.130	0.090	0.101	0.124	0.109
	LAML					0.098	0.102	0.138	0.09
	LUAD						0.119	0.097	0.089
	LUSC							0.115	0.114
	OV								0.132
B3	BLCA	0.034	0.026	0.01	0.024	0.02	0.046	0.039	0.038
	BRCA		0.012	0.014	0.011	0.026	0.016	0.041	0.027
	GBM			0.015	0.033	0.008	0.042	0.068	0.000
	HNSC				0.000	0.000	0.038	0.018	0.017
	LAML					0.084	0.015	0.03	0.063
	LUAD						0.039	0.052	0.028
	LUSC							0.057	0.018
	OV								0.073

**Table A2:** Joint analysis: relative Euclidean distances between estimated coefficient matrices.

Approach		BRCA	GBM	HNSC	LAML	LUAD	LUSC	OV	PAAD
B1	BLCA	2.108	2.090	2.503	1.943	1.994	2.104	2.122	2.081
	BRCA		2.262	2.164	2.063	2.04	2.787	2.474	1.949
	GBM			2.454	2.082	2.002	2.266	2.001	2.230
	HNSC				2.331	2.538	3.571	2.846	1.960
	LAML					2.047	2.383	2.114	2.079
	LUAD						2.250	1.958	2.147
	LUSC							2.093	2.878
	OV								2.481
B2	BLCA	1.983	1.931	1.973	1.794	1.807	2.122	1.908	1.771
	BRCA		1.928	1.891	1.963	2.063	2.423	2.093	1.906
	GBM			1.838	1.890	1.921	1.986	1.967	1.875
	HNSC				1.965	2.098	2.371	2.095	1.832
	LAML					1.843	1.889	1.866	1.940
	LUAD						2.012	1.953	1.880
	LUSC							2.064	2.351
	OV								2.071
B3	BLCA	3.664	2.176	1.992	2.251	2.052	3.432	2.185	2.049
	BRCA		2.672	3.223	2.528	3.074	1.994	2.672	3.829
	GBM			2.049	2.029	2.029	2.759	2.017	2.219
	HNSC				2.124	2.004	3.088	2.099	2.040
	LAML					1.994	2.455	1.978	1.907
	LUAD						2.998	1.983	2.101
	LUSC							2.720	3.722
	OV								2.421

**Table A3:** Data-based simulation: average true positive rates (TPRs) and false positive rates (FPRs) of different approaches, and numbers of identified true positives associated with all nine cancer types (NG).

p	Scenario		A1	A2	A3	B1	B2	B3
200	I	TPR	0.980	0.951	0.944	0.838	0.880	0.688
		FPR	0.258	0.185	0.641	0.087	0.085	0.200
		NG	7.0	8.4	3.8	5.7	8.8	1.4
	II	TPR	0.697	0.681	0.678	0.735	0.691	0.533
		FPR	0.263	0.172	0.537	0.231	0.169	0.347
		NG	4.4	3.7	0.4	4.6	4.0	0.0
	III	TPR	0.841	0.801	0.752	0.821	0.813	0.565
		FPR	0.258	0.297	0.303	0.312	0.321	0.422
		NG	7.0	6.0	5.7	5.6	6.3	1.4
	IV	TPR	0.843	0.741	0.621	0.897	0.766	0.662
		FPR	0.124	0.176	0.195	0.072	0.053	0.052
		NG	3.3	2.3	0.0	5.0	3.0	2.1
500	I	TPR	0.922	0.911	0.844	0.933	0.844	0.688
		FPR	0.248	0.152	0.452	0.114	0.122	0.173
		NG	5.0	8.0	3.0	5.0	5.0	0.0
	II	TPR	0.672	0.664	0.653	0.647	0.643	0.647
		FPR	0.191	0.171	0.165	0.025	0.063	0.128
		NG	4.7	2.8	0.4	3.8	3.1	0.0
	III	TPR	0.774	0.723	0.445	0.811	0.784	0.644
		FPR	0.173	0.160	0.107	0.173	0.053	0.181
		NG	4.0	6.3	0.0	6.2	4.8	1.2
	IV	TPR	0.822	0.678	0.617	0.864	0.719	0.646
		FPR	0.058	0.054	0.116	0.042	0.046	0.038
		NG	4.6	2.6	0.0	5.0	3.2	1.8
1000	I	TPR	0.733	0.722	0.623	0.622	0.688	0.591
		FPR	0.198	0.173	0.350	0.001	0.056	0.064
		NG	5.0	6.0	3.0	3.0	3.0	0.0
	II	TPR	0.674	0.643	0.622	0.689	0.689	0.611
		FPR	0.161	0.075	0.136	0.011	0.108	0.061
		NG	2.1	4.0	0.0	2.0	3.0	0.0
	III	TPR	0.664	0.667	0.624	0.692	0.677	0.564
		FPR	0.038	0.069	0.297	0.096	0.043	0.076
		NG	4.0	6.4	0.6	5.2	5.4	0.4
	IV	TPR	0.722	0.644	0.622	0.855	0.711	0.699
		FPR	0.093	0.100	0.136	0.016	0.015	0.009
		NG	5.0	5.0	0.0	5.0	3.0	2.0

## References

[1] Nicholson R.I., Gee J.M., Harper M.E. (2001). EGFR and cancer prognosis. Eur. J. Cancer.

[2] Petitjean A., Achatz M.I., Borresen-Dale A.L., Hainaut P., Olivier M. (2007). TP53 mutations in human cancers: functional selection and impact on cancer prognosis and outcomes. Oncogene.

[3] Gao J., Aksoy B.A., Dogrusoz U., Dresdner G., Gross B., Sumer S.O., Sun Y., Jacobsen A., Sinha R., Larsson E. (2013). Integrative analysis of complex cancer genomics and clinical profiles using the cBioPortal. Sci. Signal..

[4] Chiu C.G., Nakamura Y., Chong K.K., Huang S.K., Kawas N.P., Triche T., Elashoff D., Kiyohara E., Irie R.F., Morton D.L. (2014). Genome-wide characterization of circulating tumor cells identifies novel prognostic genomic alterations in systemic melanoma metastasis. Clin. Chem..

[5] Hoadley K.A., Yau C., Hinoue T., Wolf D.M., Lazar A.J., Drill E., Shen R., Taylor A.M., Cherniack A.D., Thorsson V. (2018). Cell-of-Origin patterns dominate the molecular classification of 10,000 tumors from 33 types of cancer. Cell.

[6] Vogelstein B., Kinzler K.W. (2004). Cancer genes and the pathways they control. Nat. Med..

[7] Easton D.F., Ford D., Bishop D.T. (1995). Breast and ovarian cancer incidence in BRCA1-mutation carriers. Breast Cancer Linkage Consortium. Am. J. Hum. Genet..

[8] Jin G., Kim M.J., Jeon H.S., Choi J.E., Kim D.S., Lee E.B., Cha S.I., Yoon G.S., Kim C.H., Jung T.H. (2010). PTEN mutations and relationship to EGFR, ERBB2, KRAS, and TP53 mutations in non-small cell lung cancers. Lung Cancer.

[9] Hammerman P.S., Sos M.L., Ramos A.H., Xu C., Dutt A., Zhou W., Brace L.E., Woods B.A., Lin W., Zhang J. (2011). Mutations in the DDR2 kinase gene identify a novel therapeutic target in squamous cell lung cancer. Cancer Discov..

[10] Dutt A., Ramos A.H., Hammerman P.S., Mermel C., Cho J., Sharifnia T., Chande A., Tanaka K.E., Stransky N., Greulich H. (2011). Inhibitor-sensitive FGFR1 amplification in human non-small cell lung cancer. PLoS ONE.

[11] Cava C., Bertoli G., Colaprico A., Olsen C., Bontempi G., Castiglioni I. (2018). Integration of multiple networks and pathways identifies cancer driver genes in pan-cancer analysis. BMC Genom..

[12] Yu X., Lian B., Wang L., Zhang Y., Dai E., Meng F., Liu D., Wang S., Liu X., Wang J. (2014). The pan-cancer analysis of gene expression patterns in the context of inflammation. Mol. Biosyst..

[13] Sharma A., Jiang C., De S. (2018). Dissecting the sources of gene expression variation in a pan-cancer analysis identifies novel regulatory mutations. Nucleic Acids Res..

[14] Martinez-Ledesma E., Verhaak R.G., Trevino V. (2015). Identification of a multi-cancer gene expression biomarker for cancer clinical outcomes using a network-based algorithm. Sci. Rep..

[15] Leiserson M.D., Vandin F., Wu H.T., Dobson J.R., Eldridge J.V., Thomas J.L., Papoutsaki A., Kim Y., Niu B., McLellan M. (2015). Pan-cancer network analysis identifies combinations of rare somatic mutations across pathways and protein complexes. Nat. Genet..

[16] Xing L., Lesperance M., Zhang X. (2019). Simultaneous prediction of multiple outcomes using revised stacking algorithms. Bioinformatics.

[17] Matlock K., De Niz C., Rahman R., Ghosh S., Pal R. (2018). Investigation of model stacking for drug sensitivity prediction. BMC Bioinform..

[18] Zhang D., Shen D. (2012). Multi-modal multi-task learning for joint prediction of multiple regression and classification variables in Alzheimer’s disease. NeuroImage.

[19] TruSight RNA Pan-Cancer Panel. https://support.illumina.com/sequencing/sequencing_kits/trusight-rna-pan-cancer-panel/questions.html.

[20] Huang J., Ma S., Xie H. (2006). Regularized estimation in the accelerated failure time model with high-dimensional covariates. Biometrics.

[21] Zhang Y., Dai Y., Zheng T., Ma S. (2011). Risk Factors of Non-Hodgkin Lymphoma. Expert Opin. Med. Diagn..

[22] Takebe N., Nguyen D., Yang S.X. (2014). Targeting notch signaling pathway in cancer: clinical development advances and challenges. Pharmacol. Ther..

[23] Zhao G., Liu Z., Ilagan M.X., Kopan R. (2010). Gamma-secretase composed of PS1/Pen2/Aph1a can cleave notch and amyloid precursor protein in the absence of nicastrin. J. Neurosci..

[24] Miele L., Miao H., Nickoloff B.J. (2006). NOTCH signaling as a novel cancer therapeutic target. Curr. Cancer Drug Targets.

[25] Laag E., Majidi M., Cekanova M., Masi T., Takahashi T., Schuller H.M. (2006). NNK activates ERK1/2 and CREB/ATF-1 via beta-1-AR and EGFR signaling in human lung adenocarcinoma and small airway epithelial cells. Int. J. Cancer.

[26] Furukawa T., Kanai N., Shiwaku H.O., Soga N., Uehara A., Horii A. (2006). AURKA is one of the downstream targets of MAPK1/ERK2 in pancreatic cancer. Oncogene.

[27] Yan J., Jiang N., Huang G., Tay J.L., Lin B., Bi C., Koh G.S., Li Z., Tan J., Chung T.H. (2013). Deregulated MIR335 that targets MAPK1 is implicated in poor outcome of paediatric acute lymphoblastic leukaemia. Br. J. Haematol..

[28] Wu Y., Chen Z., Ullrich A. (2003). EGFR and FGFR signaling through FRS2 is subject to negative feedback control by ERK1/2. Biol. Chem..

[29] Milde-Langosch K., Bamberger A.M., Rieck G., Grund D., Hemminger G., Muller V., Loning T. (2005). Expression and prognostic relevance of activated extracellular-regulated kinases (ERK1/2) in breast cancer. Br. J. Cancer.

[30] Li Z., Tognon C.E., Godinho F.J., Yasaitis L., Hock H., Herschkowitz J.I., Lannon C.L., Cho E., Kim S.J., Bronson R.T. (2007). ETV6-NTRK3 fusion oncogene initiates breast cancer from committed mammary progenitors via activation of AP1 complex. Cancer Cell.

[31] Bohlander S.K. (2005). ETV6: A versatile player in leukemogenesis. Semin. Cancer Biol..

[32] Liang J.Z., Li Y.H., Zhang Y., Wu Q.N., Wu Q.L. (2015). Expression of ETV6/TEL is associated with prognosis in non-small cell lung cancer. Int. J. Clin. Exp. Pathol..

[33] Restelli M., Magni M., Ruscica V., Pinciroli P., De Cecco L., Buscemi G., Delia D., Zannini L. (2016). A novel crosstalk between CCAR2 and AKT pathway in the regulation of cancer cell proliferation. Cell Death Dis..

[34] Hiraike H., Wada-Hiraike O., Nakagawa S., Koyama S., Miyamoto Y., Sone K., Tanikawa M., Tsuruga T., Nagasaka K., Matsumoto Y. (2010). Identification of DBC1 as a transcriptional repressor for BRCA1. Br. J. Cancer.

[35] Cho D., Park H., Park S.H., Kim K., Chung M., Moon W., Kang M., Jang K. (2015). The expression of DBC1/CCAR2 is associated with poor prognosis of ovarian carcinoma. J. Ovarian Res..

[36] Kim W., Jeong J.W., Kim J.E. (2014). CCAR2 deficiency augments genotoxic stress-induced apoptosis in the presence of melatonin in non-small cell lung cancer cells. Tumour Biol..

[37] Wagle S., Park S.H., Kim K.M., Moon Y.J., Bae J.S., Kwon K.S., Park H.S., Lee H., Moon W.S., Kim J.R. (2015). DBC1/CCAR2 is involved in the stabilization of androgen receptor and the progression of osteosarcoma. Sci. Rep..

[38] Derre L., Rivals J.P., Jandus C., Pastor S., Rimoldi D., Romero P., Michielin O., Olive D., Speiser D.E. (2010). BTLA mediates inhibition of human tumor-specific CD8+ T cells that can be partially reversed by vaccination. J. Clin. Investig..

[39] Haymaker C., Wu R., Bernatchez C., Radvanyi L. (2012). PD-1 and BTLA and CD8(+) T-cell “exhaustion” in cancer: “Exercising” an alternative viewpoint. Oncoimmunology.

[40] Fu Z., Li D., Jiang W., Wang L., Zhang J., Xu F., Pang D., Li D. (2010). Association of BTLA gene polymorphisms with the risk of malignant breast cancer in Chinese women of Heilongjiang Province. Breast Cancer Res. Treat..

[41] Oguro S., Ino Y., Shimada K., Hatanaka Y., Matsuno Y., Esaki M., Nara S., Kishi Y., Kosuge T., Hiraoka N. (2015). Clinical significance of tumor-infiltrating immune cells focusing on BTLA and Cbl-b in patients with gallbladder cancer. Cancer Sci..

[42] Cohen-Solal K.A., Boregowda R.K., Lasfar A. (2015). RUNX2 and the PI3K/AKT axis reciprocal activation as a driving force for tumor progression. Mol. Cancer.

[43] Deng S., Wang J., Hou L., Li J., Chen G., Jing B., Zhang X., Yang Z. (2013). Annexin A1, A2, A4 and A5 play important roles in breast cancer, pancreatic cancer and laryngeal carcinoma, alone and/or synergistically. Oncol. Lett..

[44] Stadler Z.K., Salo-Mullen E., Patil S.M., Pietanza M.C., Vijai J., Saloustros E., Hansen N.A., Kauff N.D., Kurtz R.C., Kelsen D.P. (2012). Prevalence of BRCA1 and BRCA2 mutations in Ashkenazi Jewish families with breast and pancreatic cancer. Cancer.

[45] Schroder M.S., Culhane A.C., Quackenbush J., Haibe-Kains B. (2011). survcomp: An R/Bioconductor package for performance assessment and comparison of survival models. Bioinformatics.

[46] Jiang Y., Shi X., Zhao Q., Krauthammer M., Rothberg B.E., Ma S. (2016). Integrated analysis of multidimensional omics data on cutaneous melanoma prognosis. Genomics.

